# Prospective cohort study of broccoli consumption frequency and all-cause and cause-specific mortality risks

**DOI:** 10.3389/fnut.2023.1286658

**Published:** 2024-01-08

**Authors:** Xiangliang Liu, Yu Chang, Yuguang Li, Xinwei Zhang, Fangqi Li, Jia Song, Hanping Shi, Xiao Chen, Jiuwei Cui

**Affiliations:** ^1^The First Hospital of Jilin University, Changchun, China; ^2^Department of Gastrointestinal Surgery/Clinical Nutrition, Beijing Shijitan Hospital, Capital Medical University, The 9th Clinical College, Beijing, China

**Keywords:** broccoli, mortality, risk factors, NHANES, diet

## Abstract

**Background:**

Broccoli is rich in vitamins, minerals, and antioxidants with broad health benefits, but its intake frequency and dose–response relationship with mortality risk remain unclear.

**Methods:**

Using data from the U.S. National Health and Nutrition Examination Survey 2003–2006, 12,486 adults were included. Broccoli intake frequency was evaluated by a food frequency questionnaire, and all-cause and cause-specific mortality risks were followed up. The relationship between broccoli intake and mortality risk was analyzed using Cox models.

**Results:**

Compared with never consumption of broccoli, different frequencies of broccoli consumption were associated with significantly decreased risks of all-cause mortality (*p* for trend <0.001). Consuming broccoli 1–2 times per week was associated with a 32–43% lower mortality risk. More frequent broccoli consumption was negatively correlated with cardiovascular and cancer mortality risks (*p* < 0.05). Consuming broccoli 1–2 times per week for males and ≥ 3 times per week for females could significantly reduce all-cause mortality risk.

**Conclusion:**

Moderate and frequent consumption of broccoli may reduce the risks of all-cause and cause-specific mortality. Optimal intake frequencies may differ by gender.

## 1 Introduction

With changes in lifestyle, the incidence and mortality of chronic non-communicable diseases such as cardiovascular disease and cancer continue to increase, posing a major threat to public health worldwide (1). Fruits and vegetables as natural health foods are nutritious and pharmacologically valuable in preventing many chronic diseases. In recent years, more and more epidemiological studies have begun to focus on the health benefits of single vegetables (2). Among them, broccoli from cruciferous vegetables has received much attention due to its unique nutritional composition and abundant bioactive components (3). Broccoli is not only rich in vitamins (vitamin C, carotenoids, etc.), minerals, and dietary fibers but also contains abundant sulfur-containing bioactive compounds (4). Numerous studies have shown that broccoli has broad-spectrum pharmacological properties such as anti-inflammatory, antioxidant, anticancer, blood pressure lowering, hypoglycemic, and hypolipidemic effects and play an important role in preventing cardiovascular diseases, cancers, type 2 diabetes, and other diseases (5–8).

For example, broccoli is abundant in sulfur-containing organic compounds, among which the compound isothiocyanate sulforaphane has been found to have certain preventive effects on prostate cancer, pancreatic cancer, leukemia, and colon cancer (9–12). However, conclusions from different studies on the dose–response relationship between broccoli intake and the prevention of chronic diseases are inconsistent. Few prospective cohort studies have found that a higher intake of cruciferous vegetables is associated with a lower risk of overall mortality and cardiovascular disease mortality compared to a lower intake of cruciferous vegetables (13). However, the available data do not provide specific recommendations for broccoli consumption frequency. Therefore, it is of great significance to use large sample prospective study results to analyze the dose–response relationship between broccoli intake frequency and all-cause and cause-specific mortality, which can provide guidance for dietary adjustment in different populations, especially dietary interventions for patients with chronic diseases.

This study utilized the prospective cohort data of the National Health and Nutrition Examination Survey (NHANES) from 2003 to 2006 to assess the relationship between different frequencies of broccoli intake and all-cause mortality and cause-specific mortality from cardiovascular diseases, cancers, and other diseases in approximately 12,486 adult participants. The results can provide a basis for developing scientific dietary guidelines and guiding dietary adjustments for patients with chronic diseases.

## 2 Methods

### 2.1 Study population

NHANES is an important cross-sectional survey conducted by the National Center for Health Statistics (NCHS) using a stratified and probabilistic random sampling design, aiming to assess the health and nutritional status of the American population. NHANES primarily acquires data via face-to-face interviews, physical examinations, and laboratory tests administered by researchers. Within the database, we initially screened 12,486 patients and subsequently subjected them to inclusion and exclusion criteria. The inclusion criteria were as follows: (1) all participants aged 20 years or older who consistently participated in NHANES cycles from 2003 to 2006 and (2) participants who cooperated with follow-up assessments and provided informed consent. The exclusion criteria were as follows: (1) age below 20 years; (2) absence of dietary data at survey initiation; (3) unavailability of survival status; (4) the outcome event was death but the cause was missing; and (5) suffering from a certain non-communicable disease at the start of follow-up. As de-identified and publicly available data were utilized, institutional review board approval was not necessary.

### 2.2 Data collection

Dietary data on broccoli were obtained from the Food Frequency Questionnaire (FFQ) based on participants’ responses to the questions: “Have you ever consumed broccoli? How frequently do you consume broccoli?,” with responses recorded for never consuming broccoli and the frequency of broccoli consumption. Participants’ responses were used to classify broccoli consumption into four categories: never (“never”), infrequently (“less than once a week”), occasionally (“1 to 2 times per week but less than 3 times per week”), and regularly (“3 or more times per week”). Furthermore, covariate information such as age, gender, race, education level, poverty income ratio (PIR), body mass index (BMI), smoking status, alcohol consumption status, and history of hypertension, dyslipidemia, and diabetes was collected through questionnaire surveys. Participants were stratified based on PIR as follows: PIR (low) ≤ 1, PIR (medium) > 1–<4, and PIR (high) ≥ 4 (14). BMI was determined as the ratio of weight (kg) to the square of height (m^2^). Smoking status was categorized as follows: “Current” referred to individuals who reported smoking more than 100 cigarettes in their lifetime and currently smoked on all or some days; “Former” referred to individuals who reported smoking more than 100 cigarettes in their lifetime but currently did not smoke; and “Never” referred to individuals who reported smoking less than 100 cigarettes in their lifetime. In terms of alcohol consumption status, “Now” was defined as participants who consumed alcoholic beverages in the past year, “Former” was defined as participants who had consumed alcoholic beverages in the past year but had stopped drinking at the time of the survey, and “Never” was defined as participants who had not consumed any alcoholic beverages in the past year. If systolic blood pressure is ≥140 mmHg or diastolic blood pressure is ≥90 mmHg, with the use of antihypertensive medication, an individual can be diagnosed with hypertension. If triglyceride level is ≥150 mg/dL; high-density lipoprotein is <140 mg/dL; and low-density lipoprotein is ≥130 mg/dL, with the use of lipid-lowering medication, an individual can be diagnosed with dyslipidemia. If fasting blood glucose level is ≥7.0 mmol/L; 2-h postprandial blood glucose is ≥11.1 mmol/L; and glycated hemoglobin is ≥6.5%, with the use of hypoglycemic medication, an individual can be diagnosed with diabetes if they meet any of the above criteria.

Blood samples were collected at the start of follow-up to obtain values for monocytes, lymphocytes, platelets, and neutrophils. Based on these values, we calculated the following inflammation-related markers: lymphocyte-to-monocyte ratio (LMR) and systemic immune-inflammation index (SII), which is calculated as platelet × neutrophil count divided by lymphocyte count; and neutrophil-to-lymphocyte ratio (NLR) (15).

The National Death Index (NDI) database was utilized to associate the information of NHANES survey participants with their corresponding death records. This approach was confined to a designated range to ensure the attainment of precise mortality data for NHANES participants. The interval from the commencement of household interviews to either loss to follow-up or death was accounted for as the follow-up duration. The NDI database was employed to acquire the mortality status. Furthermore, the specific causes of death were ascertained as delineated by the International Classification of Diseases (ICD) 10.

### 2.3 Statistical analysis

In descriptive statistics, normal continuous variables were described using the mean (standard deviation, SD). Statistical differences were described using a t-test or analysis of variance. Non-normal continuous variables were described using the median (interquartile range, IQR), and statistical differences were described using non-parametric tests. Categorical variables were described using frequency (represented as percentages), and statistical differences were described using the chi-square test. The Cox proportional hazards models were formulated to estimate hazard ratios (HRs) accompanied by 95% confidence intervals. Univariate and multivariate regression analyses were employed to evaluate the association between various determinants, notably broccoli intake frequency and the risks associated with all-cause and specific-cause mortality. A threshold of value of *p* of <0.05 was established for determining statistical significance. Data preprocessing and statistical evaluations were executed utilizing the intricate survey design module of the SPSS 25.0 (IBM) statistical software. Forest plots along with the correlation matrix were generated using R software version 4.2.3.

## 3 Results

### 3.1 Baseline characteristics

**Figure 1:**
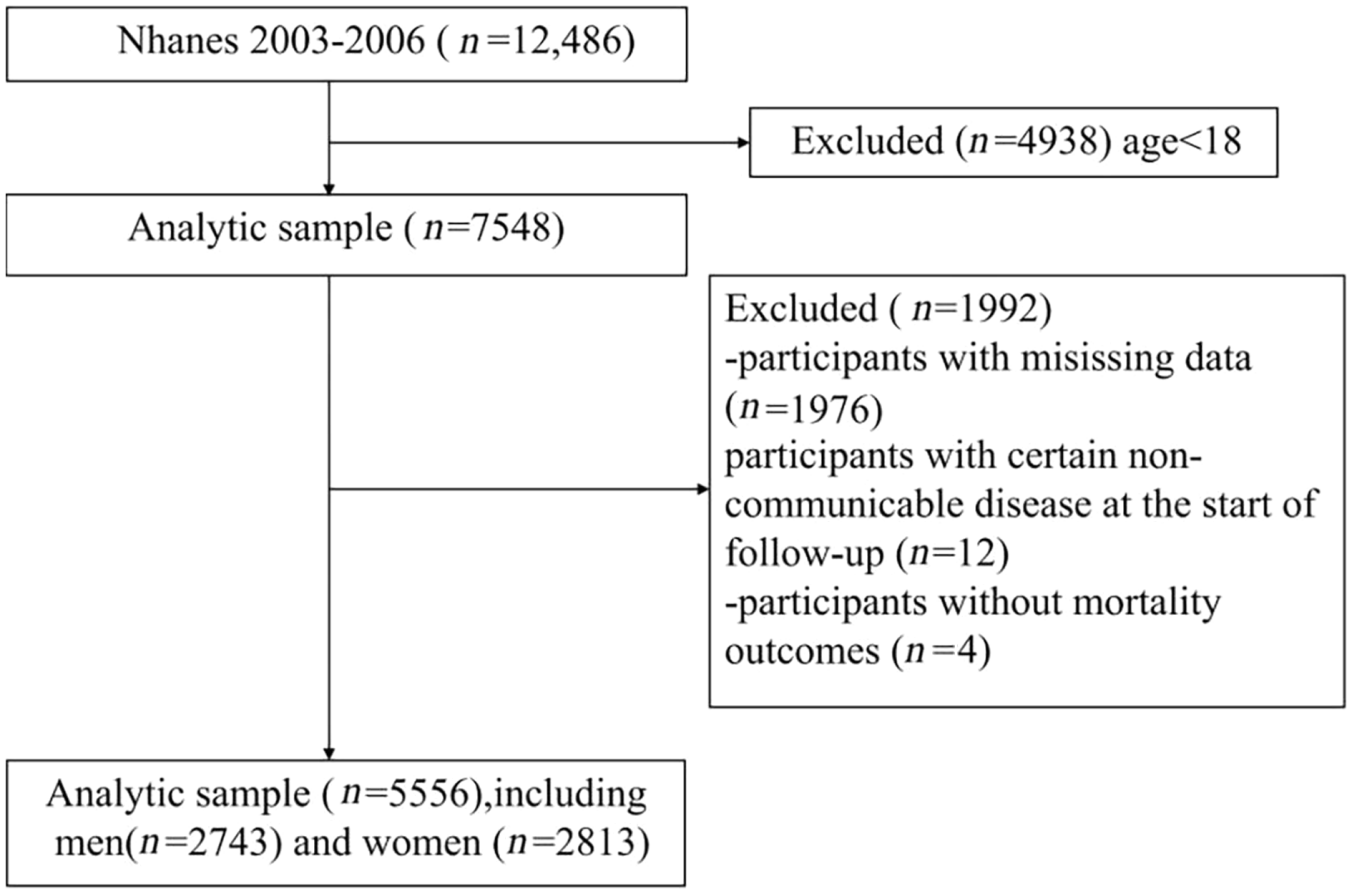
Flowchart of the study and participants excluded from the study.

Our study ultimately encompassed 5,556 adults aged 20 years and older who provided valid responses to the broccoli diet query in the NHANES Food Frequency Questionnaire. The sample included 2,743 males and 2,813 females. Detailed specifics can be found in Figure 1. During the follow-up period that ended on 31 December 2019, the overall count of deaths from all causes reached 1,405, which represented 25.3% of the total sample population. The causes of these deaths, categorized according to the ICD-10, were primarily distributed across seven categories: cardiovascular diseases, pulmonary and chronic respiratory diseases, renal diseases, malignant tumors, diabetes, accidents, and miscellaneous causes. These categories also include the following distribution: Cardiovascular disease was responsible for 504 deaths, representing 35.9% of the total deaths; malignant tumors accounted for 292 deaths, representing 20.7% of the total deaths; 107 deaths were due to pulmonary and chronic respiratory diseases, representing 7.6% of the total deaths; diabetes resulted in 49 deaths, totaling 3.5% of all deaths; renal diseases led to 36 deaths, representing 2.6% of total deaths; 38 deaths were caused by accidents, accounting for 2.7% of all deaths; and 379 deaths were attributed to miscellaneous causes, representing 27% of all deaths.

As shown in Table 1, an observed disparity in the frequency of broccoli consumption between the genders indicated a more prevalent intake among females than males. A variance in broccoli consumption frequency was also detected across racial demographics, with the non-Hispanic white population reported to have more frequent consumption compared to other races. Discrepancies in broccoli consumption frequency were apparent among distinct PIR groups. Those falling within the medium-range PIR category (PIR > 1–<4) exhibited greater frequency compared to both the low (≤1) and high PIR groups (≥4). Frequent broccoli consumers demonstrated a decreased frequency of consumption in smokers compared to non-smokers. This implies that smoking habits could potentially impinge on regular broccoli consumption. Among habitual broccoli consumers, individuals who regularly consumed alcohol were observed to consume broccoli with greater frequency than those who had ceased alcohol consumption or abstained from it altogether, suggesting that alcohol consumption may be linked to an increased frequency of broccoli consumption.

**Table 1:** Baseline characteristic variables according to the broccoli consumption frequency.

Variable	Total (*n* = 5,556)	0 (*n* = 739)	1 (*n* = 3,181)	2 (*n* = 1,216)	3 (*n* = 420)	Statistic	*p*
Age, *n* (%)						χ^2^ = 5.434	0.143
<65	3,893 (70.07)	491 (66.44)	2,249 (70.70)	855 (70.31)	298 (70.95)		
≥65	1,663 (29.93)	248 (33.56)	932 (29.30)	361 (29.69)	122 (29.05)		
Gender, *n* (%)						χ^2^ = 117.569	<0.001
Male	2,743 (49.37)	486 (65.76)	1,567 (49.26)	531 (43.67)	159 (37.86)		
Female	2,813 (50.63)	253 (34.24)	1,614 (50.74)	685 (56.33)	261 (62.14)		
Race, *n* (%)						χ^2^ = 47.293	<0.001
Race (1)	3,149 (56.68)	410 (55.48)	1812 (56.96)	715 (58.80)	212 (50.48)		
Race (2)	1,067 (19.2)	150 (20.30)	625 (19.65)	184 (15.13)	108 (25.71)		
Race (3)	989 (17.8)	148 (20.03)	560 (17.60)	208 (17.11)	73 (17.38)		
Race (4)	351 (6.32)	31 (4.19)	184 (5.78)	109 (8.96)	27 (6.43)		
PIR, *n* (%)						χ^2^ = 109.197	<0.001
PIR (1)	860 (15.48)	158 (21.38)	467 (14.68)	162 (13.32)	73 (17.38)		
PIR (2)	3,125 (56.25)	469 (63.46)	1820 (57.21)	616 (50.66)	220 (52.38)		
PIR (3)	1,571 (28.28)	112 (15.16)	894 (28.10)	438 (36.02)	127 (30.24)		
Education level, *n* (%)						χ^2^ = 169.380	<0.001
Middle school or below	1,398 (25.16)	291 (39.38)	770 (24.21)	238 (19.57)	99 (23.57)		
High school	1,415 (25.47)	210 (28.42)	864 (27.16)	253 (20.81)	88 (20.95)		
College or above	2,743 (49.37)	238 (32.21)	1,547 (48.63)	725 (59.62)	233 (55.48)		
Diabetes, *n* (%)						χ^2^ = 9.310	0.157
No	4,363 (78.53)	554 (74.97)	2,511 (78.94)	963 (79.19)	335 (79.76)		
Prophase	347 (6.25)	46 (6.22)	203 (6.38)	71 (5.84)	27 (6.43)		
Yes	846 (15.23)	139 (18.81)	467 (14.68)	182 (14.97)	58 (13.81)		
Smoke History, *n* (%)						χ^2^ = 69.519	<0.001
Never	2,765 (49.77)	320 (43.30)	1,556 (48.92)	654 (53.78)	235 (55.95)		
Former	1,596 (28.73)	202 (27.33)	904 (28.42)	354 (29.11)	136 (32.38)		
Now	1,195 (21.51)	217 (29.36)	721 (22.67)	208 (17.11)	49 (11.67)		
Alcohol History, *n* (%)						χ^2^ = 37.452	<0.001
Never	750 (13.5)	112 (15.16)	410 (12.89)	151 (12.42)	77 (18.33)		
Former	1,254 (22.57)	205 (27.74)	689 (21.66)	251 (20.64)	109 (25.95)		
Now	3,552 (63.93)	422 (57.10)	2082 (65.45)	814 (66.94)	234 (55.71)		
Hyperlipidemia, *n* (%)						χ^2^ = 4.195	0.241
No	1,494 (26.89)	189 (25.58)	840 (26.41)	337 (27.71)	128 (30.48)		
Yes	4,062 (73.11)	550 (74.42)	2,341 (73.59)	879 (72.29)	292 (69.52)		
Hypertension, *n* (%)						χ^2^ = 6.521	0.089
No	3,043 (54.77)	378 (51.15)	1742 (54.76)	694 (57.07)	229 (54.52)		
Yes	2,513 (45.23)	361 (48.85)	1,439 (45.24)	522 (42.93)	191 (45.48)		
BMI, M (Q₁, Q₃)	27.73 (24.24–31.96)	27.66 (24.26–31.95)	27.95 (24.46–32.20)	27.40 (24.00–31.57)	27.15 (23.89–31.71)	χ^2^ = 7.032	0.071

Race (1): Non-Hispanic white, Race (2): Non-Hispanic Black, Race (3): Mexican-American, Race (4): Other.

PIR (1):≤1, PIR (2):>1–<4, PIR (3):≥4.

### 3.2 Univariate and multivariate analyses of factors associated with all-cause and cause-specific mortality risks

**Figure 2:**
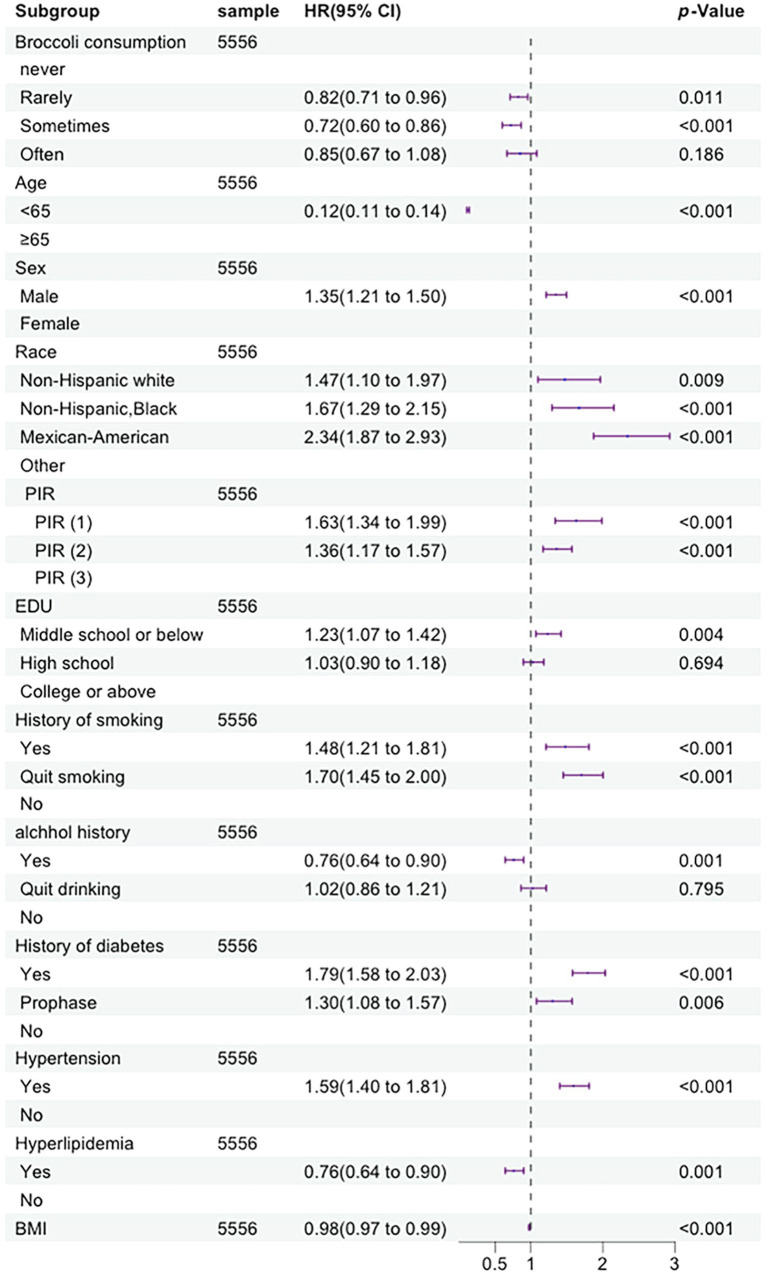
Forest plot displaying the hazard ratios of all-cause mortality by multivariate Cox regression analysis (By R 4.2.3).

In this cohort, we analyzed a range of variables, including age, gender, race, poverty income ratio (PIR), education level, smoking status, drinking status, history of hypertension, dyslipidemia, and diabetes. In the univariate Cox proportional hazards regression model, we identified 12 indicators that were significantly associated with the risk of all-cause mortality. These indicators include age, gender, race, poverty income ratio (PIR), education level, smoking status, drinking status, history of hypertension, history of dyslipidemia, history of diabetes, broccoli consumption frequency, and BMI. The multivariate Cox proportional hazards regression model confirmed that the same 12 indicators identified in the univariate analysis remain significantly associated with the risk of all-cause mortality. Specific details can be seen in Figure 2.

**Figure 3:**
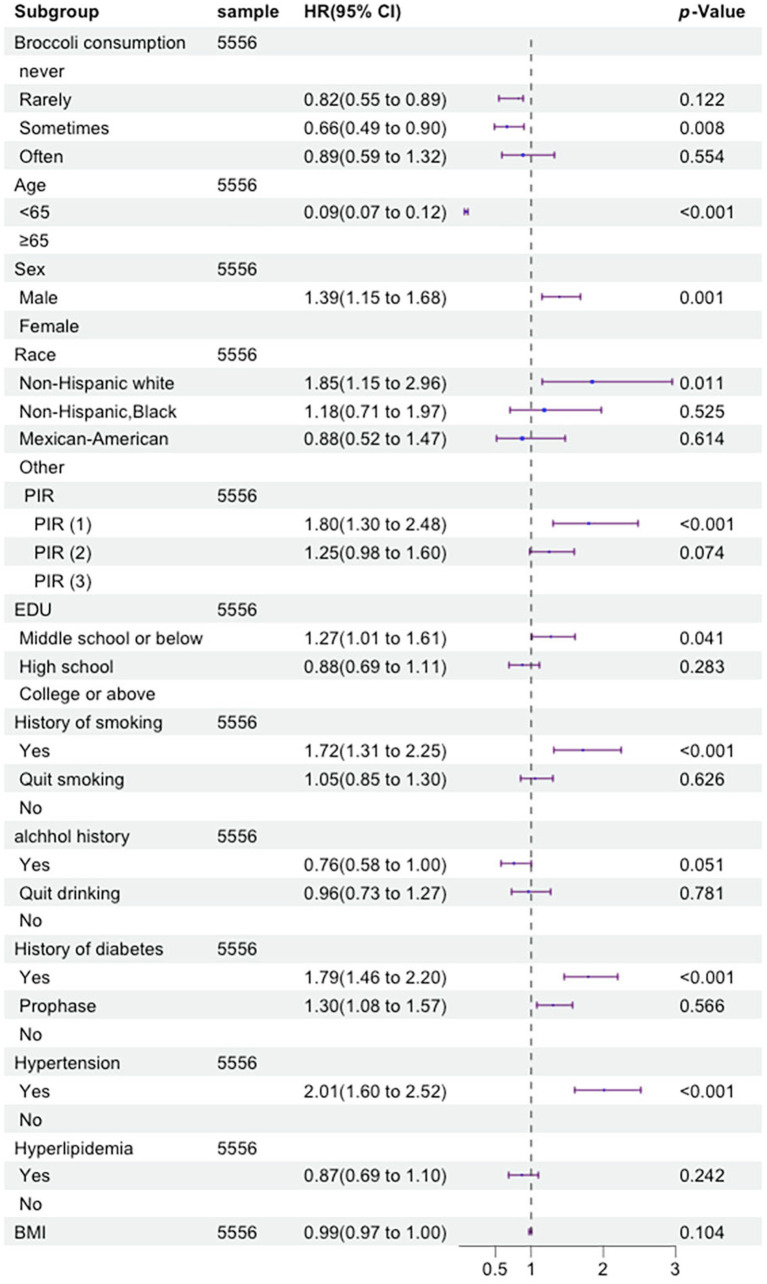
Forest plot displaying the hazard ratios of cardiovascular and cerebrovascular disease mortality by multivariate Cox regression analysis (By R 4.2.3).

The univariate Cox proportional hazards regression model identified 11 indicators that were significantly associated with the risk of cardiovascular mortality. These indicators include age, gender, race, PIR, education level, smoking status, drinking status, history of hypertension, history of dyslipidemia, history of diabetes, and broccoli consumption frequency. The multivariate Cox proportional hazards regression model confirmed that nine indicators remained significantly associated with the risk of cardiovascular mortality. These indicators include age, gender, race, PIR, education level, smoking status, history of hypertension, history of diabetes, and broccoli consumption frequency. The univariate Cox proportional hazards regression model revealed that 10 indicators were significantly associated with the risk of cancer mortality. These indicators include age, gender, race, PIR, education level, smoking status, drinking status, history of hypertension, history of diabetes, and broccoli consumption frequency. The multivariate Cox proportional hazards regression model demonstrated that eight indicators remained significantly associated with the risk of cancer mortality. These indicators include age, race, PIR, education level, history of hypertension, history of dyslipidemia, broccoli consumption frequency, and BMI. Specific details can be seen in Figures 3 4.

**Figure 4:**
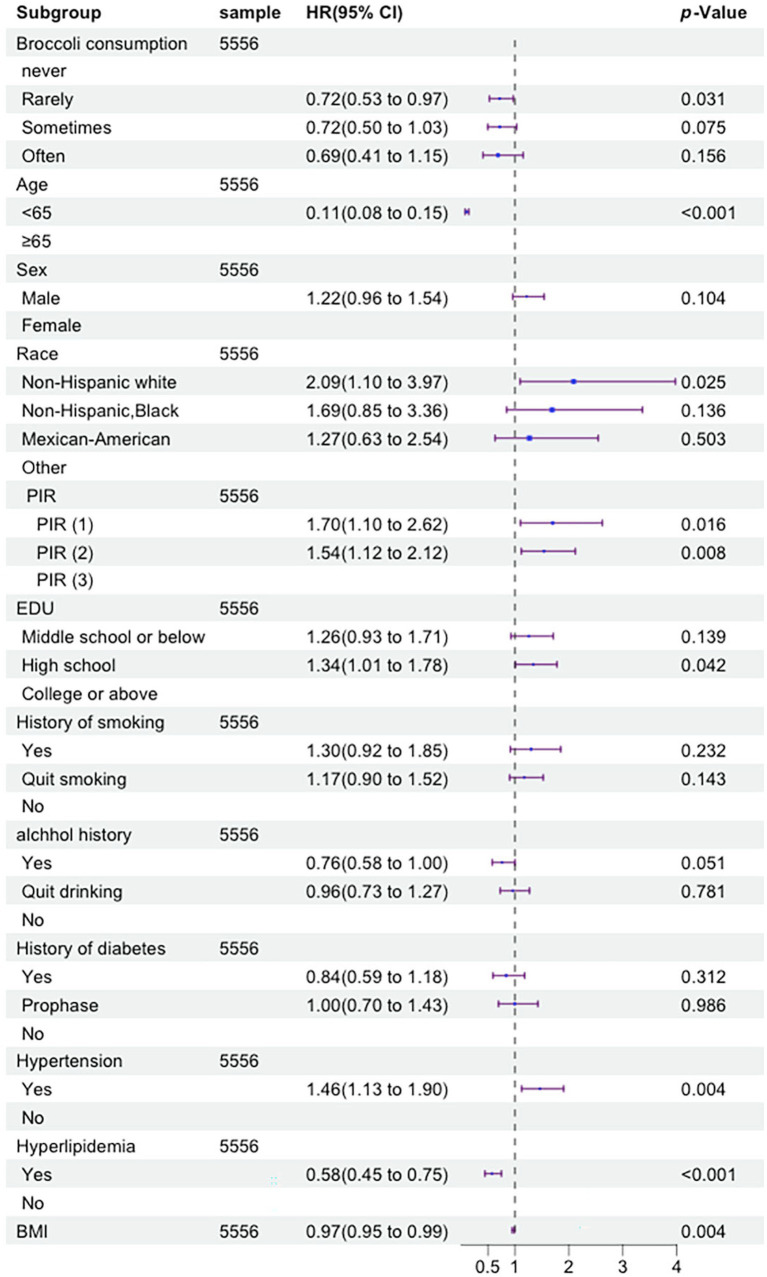
Forest plot displaying the hazard ratios of tumor mortality by multivariate Cox regression analysis (By R 4.2.3).

### 3.3 Association between broccoli consumption frequency and risks of all-cause and cause-specific mortality after model adjustment

Given the potential confounding effects of covariates on outcomes, we employed adjusted models to reassess the relationship between the frequency of broccoli consumption and the risks of all-cause mortality, as well as specific diseases such as cardiovascular mortality and cancer mortality. We employed three adjustment models to control for potential confounders. Model 1 was adjusted for age, gender, and race; Model 2 was adjusted for age, gender, race, PIR, education level, and BMI; and Model 3 was adjusted for age, gender, race, PIR, education level, BMI, smoking status, drinking status, history of hypertension, history of dyslipidemia, and history of diabetes. In the analysis of model-adjusted results regarding the association between broccoli consumption frequency and the risk of all-cause mortality, the rarely broccoli consumption group (“less than once a week”) exhibited a mortality risk hazard ratio (HR) (95% confidence interval [CI]) of 0.700 (0.605–0.809) in Model 1, 0.785 (0.677–0.909) in Model 2, and 0.825 (0.711–0.957) in Model 3, compared to the reference group of individuals who never consume broccoli. For the sometimes broccoli consumption group (“1 to 2 times per week but less than 3 times per week”), the mortality risk HR (95% CI) was 0.577 (0.484–0.689) in Model 1 and 0.680 (0.568–0.815) in Model 2. The mortality risk HR (95% CI) for the often broccoli consumption group (“3 or more times per week”) was 0.705 (0.555–0.895) in Model 1 and 0.723 (0.603–0.865) in Model 2 (Supplementary Figure S1).

In the analysis of model-adjusted results regarding the association between broccoli consumption frequency and the risk of cardiovascular mortality, the rarely broccoli consumption group (“less than once a week”) exhibited a mortality risk hazard ratio (HR) (95% CI) of 0.688 (0.541–0.874) in Model 1 and 0.779 (0.611–0.995) in Model 2 compared to the reference group of individuals who never consume broccoli. For the sometimes broccoli consumption group (“1 to 2 times per week but less than 3 times per week”), the mortality risk hazard ratio (HR) (95% CI) was 0.522 (0.388–0.702) in Model 1, 0.619 (0.457–0.839) in Model 2, and 0.664 (0.490–0.900) in Model 3 (Supplementary Figure S2).

In the analysis of the model-adjusted results regarding the association between broccoli consumption frequency and cancer mortality risk, compared to the reference group of individuals who never consume broccoli, the rarely broccoli consumption group (“less than once a week”) exhibited a mortality risk hazard ratio (HR) (95% CI) of 0.622 (0.461–0.838) in Model 1, 0.697 (0.515–0.944) in Model 2, and 0.716 (0.528–0.970) in Model 3. The sometimes broccoli consumption group (“1 to 2 times per week but less than 3 times per week”) had a mortality risk HR (95% CI) of 0.587 (0.411–0.837) in Model 1. The often broccoli consumption group (“3 or more times per week”) had a mortality risk HR (95% CI) of 0.599 (0.359–0.998) in Model 1 (Supplementary Figure S3).

### 3.4 Association between broccoli consumption frequency and all-cause mortality risk after model adjustment stratified by gender

The distribution of broccoli consumption frequency exhibited gender differences, as indicated in Table 1. Thus, we conducted gender-stratified analyses and adjusted for confounding factors to more precisely assess the relationship between broccoli consumption frequency and the risk of all-cause mortality in each gender population. Among men, individuals in the rarely broccoli consumption group (“less than once a week”) had a hazard ratio (HR) (95% CI) of 0.650 (0.544–0.776) in Model 1 (adjusted for age, gender, and race), 0.719 (0.599–0.862) in Model 2 (adjusted for variables in Model 1 plus poverty income ratio, education level, and BMI), and 0.743 (0.619–0.892) in Model 3 (adjusted for variables in Models 1 and 2 plus smoking status, drinking status, history of hypertension, history of dyslipidemia, and history of diabetes) compared to those who never consumed broccoli. Among males, individuals who occasionally consumed broccoli (“1 to 2 times per week but less than 3 times per week”) had a hazard ratio (HR) (95% CI) of 0.560 (0.445–0.705) in Model 1, 0.651 (0.514–0.825) in Model 2, and 0.657 (0.519–0.832) in Model 3. These findings suggest a more pronounced reduction in the risk of all-cause mortality among males who consume broccoli occasionally.

Among females, individuals in the sometimes broccoli consumption group (“1 to 2 times per week but less than 3 times per week”) had a hazard ratio (HR) (95% CI) of 0.634 (0.473–0.849) in Model 1 and 0.742 (0.552–0.997) in Model 2 compared to those who never consumed broccoli. The HR (95% CI) of the often broccoli consumption group (“3 or more times per week”) was 0.627 (0.431–0.913) in Model 1 and 0.678 (0.465–0.989) in Model 2. These findings suggest a more pronounced reduction in the risk of all-cause mortality among females who consume broccoli frequently. Additional information can be found in Supplementary Figure S4.

### 3.5 Association between broccoli consumption frequency and inflammation markers

**Figure 5:**
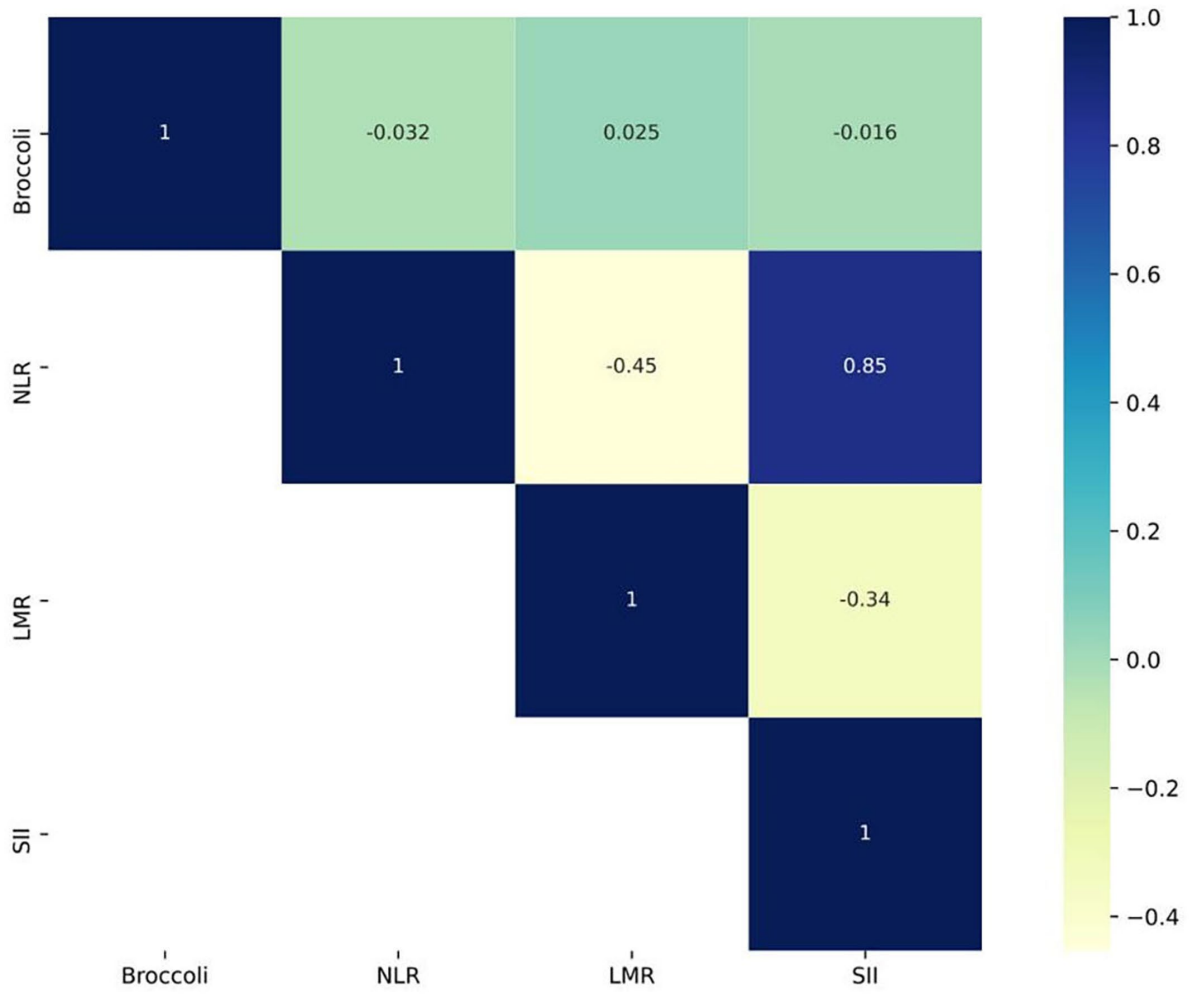
Correlation matrix plot describing the frequency of broccoli consumption and inflammatory markers (By R 4.2.3).

To investigate the relationship between the frequency of broccoli consumption and inflammation markers, we conducted a correlation analysis using a correlation matrix. The results revealed that the correlation coefficient between broccoli consumption frequency and NLR was −0.032, *p* = 0.017; the correlation coefficient between broccoli consumption frequency and LMR was 0.025, *p* = 0.063; and the correlation coefficient between broccoli consumption frequency and SII was −0.016, *p* = 0.248. These findings suggest a negative correlation between broccoli consumption frequency and NLR. More specific details can be seen in Figure 5.

## 4 Discussion

The results of this study show that, compared with those who never eat broccoli, participants who consumed broccoli at different frequencies had significantly lower all-cause mortality risks, showing a negative correlation trend, which is consistent with the results of previous meta-analyses on fruit and vegetable intake and mortality risk (16). Specifically, eating broccoli 1–2 times per week can reduce all-cause mortality risk by 32–43%. In addition, broccoli intake frequency was negatively correlated with cardiovascular mortality and cancer mortality. Considering that different genders may respond differently to broccoli intake, we conducted stratified analysis by gender and found that, compared with females, males were more inclined to eat broccoli 1–2 times per week, while females were more inclined to eat broccoli ≥3 times per week to obtain the effect of reducing all-cause mortality. Finally, we found that broccoli intake frequency was negatively correlated with the inflammatory marker NLR. Overall, this study determined a dose–response relationship between broccoli intake frequency and reduced risks of all-cause and cause-specific mortality, providing a basis for developing scientific dietary guidelines.

Our findings are validated by several previous large-scale studies, two of which were conducted in Asia, demonstrating that cruciferous vegetables can enhance cardiovascular health and reduce mortality risk. Several prospective studies have indicated that high fruit and vegetable consumption does not necessarily correlate with reduced cancer incidence. Therefore, we further explored the potential mechanisms through which consuming broccoli may lower the risk of mortality, taking into account its anti-inflammatory and antioxidant properties, its role in modulating lipid metabolism, and its influence on blood glucose homeostasis.

Broccoli contains abundant direct antioxidant nutrients such as vitamin C, carotenoids, and anthocyanins (7), which can eliminate excessive active oxygen-free radicals and inhibit oxidative stress in the body. In addition, sulfur-containing phytochemicals are unique bioactive components in broccoli, such as glucosinolate, which can exert anti-inflammatory effects by inhibiting the production and release of inflammatory factors (17), as well as by protecting the body from inflammatory damage by stimulating the production of antioxidant enzymes (18). Substantial evidence indicates that chronic inflammation and oxidative stress play important roles in the pathogenesis of various chronic diseases, including cancers, cardiovascular diseases, and type 2 diabetes (19). Chronic inflammation can lead to the release of additional cytokines and growth factors, stimulating angiogenesis and cell proliferation, degrading extracellular matrix enzymes, and providing an advantageous environment for tumor growth (20). Moreover, chronic inflammation can also result in endothelial cell oxidative stress, promote platelet activation, cause endothelial cell damage, and can be considered a key driving factor in the development of cardiovascular diseases (8). Therefore, the abundant anti-inflammatory and antioxidant components in broccoli may be important nutritional mechanisms for its beneficial effects against chronic diseases.

Broccoli contains abundant vitamin K1, which is a fat-soluble vitamin that can promote the generation of clotting factors and inhibit the synthesis and secretion of cholesterol in the liver (21). In addition, minerals in broccoli, such as magnesium, can also reduce cholesterol absorption. The flavonoids abundant in broccoli are believed to have the ability to scavenge free radicals and accelerate cholesterol breakdown (22). The rich dietary fiber, especially the soluble mucilaginous dietary fiber in broccoli, can bind with bile acids to form insoluble complexes, increasing bile acid excretion in feces and thus reducing bile acid reabsorption and decreasing cholesterol synthesis in the body (23). By regulating lipid metabolism, broccoli may reduce the incidence of cardiovascular disease.

**Figure 6:**
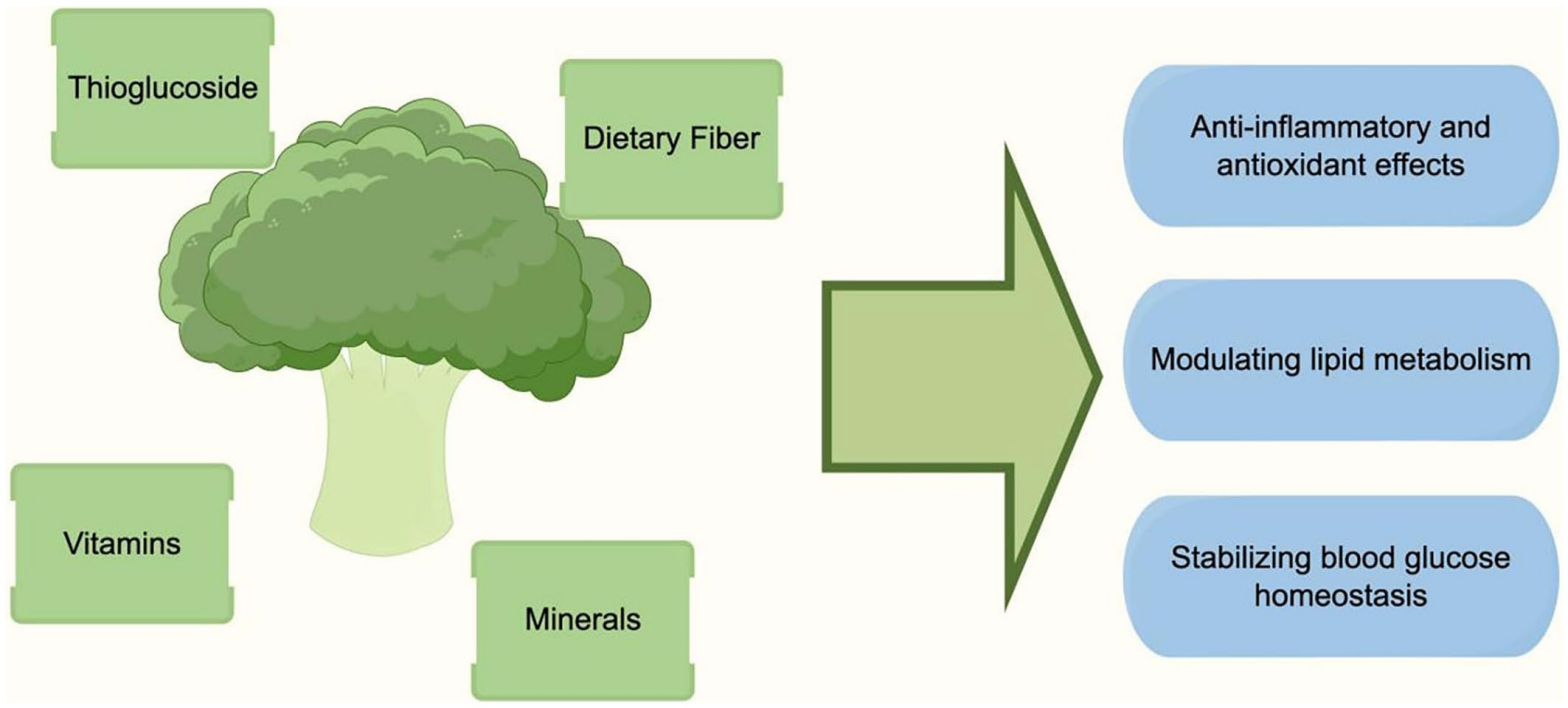
Potential mechanisms of broccoli intake in reducing mortality risk (By Figdraw).

The rich dietary fiber, especially water-soluble fiber in broccoli, can prolong the retention time of food in the gastrointestinal tract and slow down the digestion and absorption of carbohydrates, thereby inhibiting drastic fluctuations in postprandial blood glucose and insulin levels (24). In addition, sulforaphane in broccoli can also promote insulin signal transduction in skeletal muscle and liver tissues, enhancing insulin sensitivity in insulin-resistant tissues and thus lowering blood glucose (25). The high content of vitamin C in broccoli is capable of protecting pancreatic beta cells from oxidative stress damage. Adequate intake of broccoli helps maintain the functionality of pancreatic beta cells, preventing insufficient insulin secretion due to pancreatic cell injury and thus stabilizing blood glucose regulation (22). By regulating glucose and lipid metabolism and enhancing insulin sensitivity, broccoli can reduce the risks of metabolic syndrome, type 2 diabetes, and cardiovascular diseases. The mechanism of broccoli intake in reducing mortality risk, as illustrated above, can be seen in Figure 6.

This study found that, compared with females, males were more suitable to eat broccoli 1–2 times per week, while females were more suitable to eat broccoli ≥3 times per week to obtain the effect of reducing all-cause mortality. This may be related to gender differences in dietary patterns and physiological metabolism. On the one hand, the intake of fruits and vegetables is inherently higher in females’ diets (26), so they may need to eat broccoli more frequently to produce significant health effects. On the other hand, increased estrogen levels can promote the expression of glutathione peroxidase, enhancing the body’s antioxidant capacity (27), which may make females require higher amounts of antioxidant nutrients in broccoli. Overall, there may be some differences in the optimal frequency of broccoli intake between different genders, which should be further clarified in future studies to provide a basis for dietary guidance for both genders.

This study determined the dose–response relationship between broccoli intake frequency and reduced mortality risk in a large sample population, providing an important basis for developing scientific dietary guidelines. However, as an observational study, it cannot demonstrate a direct causal relationship between broccoli and mortality risk reduction. In addition, considering that the participants were all American adults, there may be some differences between different ethnicities, so the research conclusions should be generalized with caution. Finally, FFQ assessments of dietary intake have some subjectivity and may be subject to recall bias. Future rigorous clinical trials are still needed to verify the health effects of broccoli consumption and determine appropriate intake ranges based on different populations in order to provide more rigorous evidence for formulating dietary guidelines.

## Data availability statement

The raw data supporting the conclusions of this article will be made available by the authors, without undue reservation.

## Ethics statement

The studies involving humans were approved by the National Center for Health Statistics Research Ethics Review Board, duly approved by the ethical review committee (protocol #98-12, #2005-06). The patients/participants provided their written informed consent to participate in this study.

## Author contributions

XL: Conceptualization, Data curation, Writing – original draft. YC: Formal analysis, Writing – original draft, Data curation. YL: Data curation, Visualization, Writing – original draft. XZ: Data curation, Writing – original draft. FL: Formal analysis, Investigation, Writing – original draft. JS: Supervision, Visualization, Writing – original draft. HS: Data curation, Writing – original draft. XC: Conceptualization, Supervision, Validation, Writing – review & editing. JC: Conceptualization, Supervision, Validation, Writing – review & editing.
