# Supplementary material for: Prospective cohort study of broccoli consumption frequency and all-cause and cause-specific mortality risks
Source: Front Nutr. 2024 Jan 8;10:1286658. doi: 10.3389/fnut.2023.1286658 (PMC10800680; doi:10.3389/fnut.2023.1286658)
Supplement: Supplementary file 1 [file Data_Sheet_1.pdf]

Supplementary Material

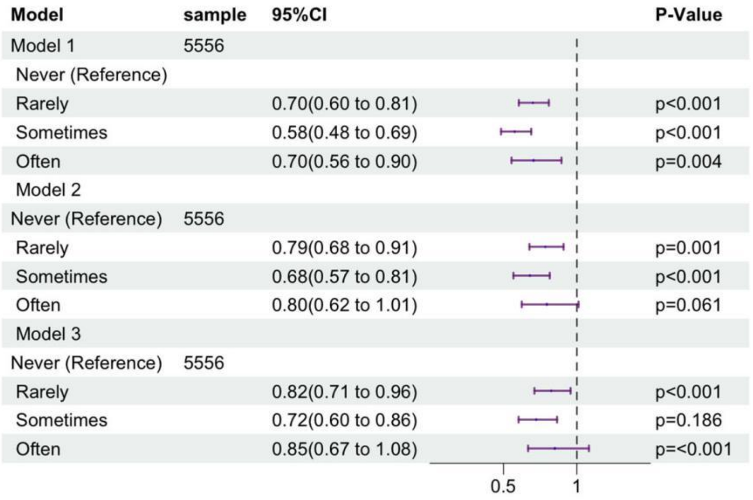

Supplementary Figure 1. The relationship between broccoli consumption frequency and all-cause mortality after model adjustment

Model	sample	95%CI	P-Value
Model 1	5556		
Never (Reference)			
Rarely		0.69(0.54 to 0.87)	p=0.002
Sometimes		0.52(0.39 to 0.70)	p<0.001
Often		0.70(0.47 to 1.04)	p=0.078
Model 2	5556		
Never (Reference)			
Rarely		0.78(0.61 to 0.99)	p=0.046
Sometimes		0.62(0.46 to 0.84)	p=0.002
Often		0.81(0.54 to 1.20)	p=0.286
Model 3	5556		
Never (Reference)			
Rarely		0.82(0.65 to 1.05)	p=0.122
Sometimes		0.66(0.49 to 0.90)	p=0.008
Often		0.89(0.59 to 1.32)	p=0.054

Supplementary Figure 2. The relationship between broccoli consumption frequency and cardiovascular and cerebrovascular disease mortality after model adjustment

Model	sample	95%CI	P-Value
Model 1	5556		
Never (Reference)			
Rarely		0.62(0.46 to 0.84)	p=0.002
Sometimes		0.59(0.41 to 0.84)	p=0.003
Often		0.60(0.36 to 1.00)	p=0.049
Model 2	5556		
Never (Reference)			
Rarely		0.70(0.52 to 0.94)	p=0.020
Sometimes		0.70(0.49 to 1.00)	p=0.053
Often		0.66(0.40 to 1.11)	p=0.118
Model 3	5556		
Never (Reference)			
Rarely		0.72(0.53 to 0.97)	p=0.031
Sometimes		0.72(0.50 to 1.03)	p=0.075
Often		0.69(0.41 to 1.15)	p=0.156

Supplementary Figure 3. The relationship between broccoli consumption frequency and tumor mortality after model adjustment

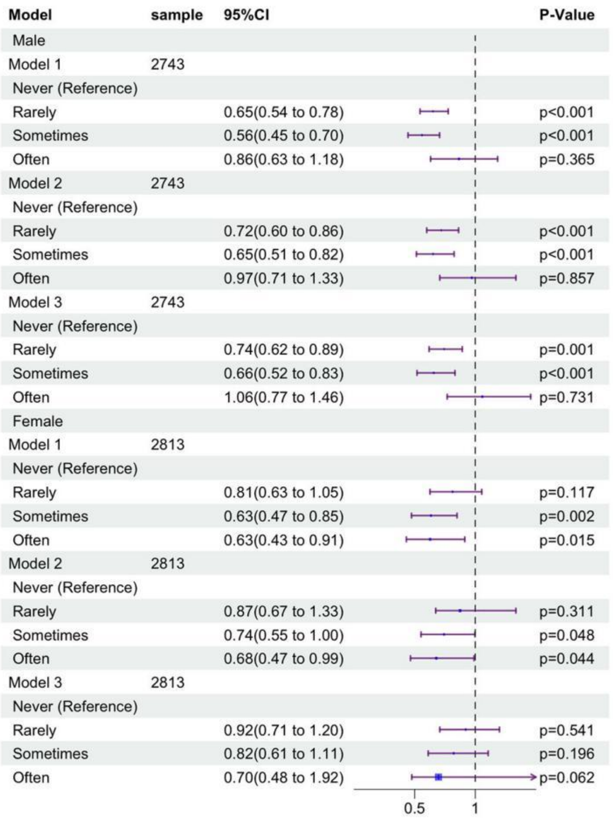

Supplementary Figure 4. The relationship between broccoli consumption frequency and all-cause mortality according to the stratification by gender after model adjustment
